# Urokinase-Type Plasminogen Activator Enhances the Neuroprotective Activity of Brain-Derived Neurotrophic Factor in a Model of Intracerebral Hemorrhage

**DOI:** 10.3390/biomedicines10061346

**Published:** 2022-06-08

**Authors:** Stalik Dzhauari, Svetlana Litvinova, Anastasia Efimenko, Natalia Aleksandrushkina, Nataliya Basalova, Maxim Abakumov, Natalia Danilova, Pavel Malkov, Vadim Balabanyan, Tatiana Bezuglova, Viktor Balayants, Maxim Mnikhovich, Mikhail Gulyaev, Mariya Skryabina, Vladimir Popov, Dmitry Stambolsky, Tatiana Voronina, Vsevolod Tkachuk, Maxim Karagyaur

**Affiliations:** 1 Faculty of Medicine, Lomonosov Moscow State University, 27/1, Lomonosovsky Ave., 119192 Moscow, Russia; stalik.djauari@yandex.ru (S.D.); efimenkoan@gmail.com (A.E.); n.alexandrushkina@gmail.com (N.A.); natalia_ba@mail.ru (N.B.); bal.pharm@mail.ru (V.B.); gulyaev@physics.msu.ru (M.G.); skrebbka@gmail.com (M.S.); galiantus@gmail.com (V.P.); tkachuk@fbm.msu.ru (V.T.); 2 Federal State Budgetary Institution “Research Zakusov Institute of Pharmacology”, 8, Baltiyskaya Str., 125315 Moscow, Russia; sa_litvinova@mail.ru (S.L.); voroninata38@gmail.com (T.V.); 3 Institute for Regenerative Medicine, Medical Research and Education Center, Lomonosov Moscow State University, 27/10, Lomonosovsky Ave., 119192 Moscow, Russia; 4 Department of Medical Nanobiotechnology, National University of Science and Technology MISiS, 4, Leninskiy Ave., 119049 Moscow, Russia; abakumov_ma@rsmu.ru; 5 Center for Precision Genome Editing and Genetic Technologies for Biomedicine, Pirogov Russian National Research Medical University, 1, Ostrovityanova Str., 117997 Moscow, Russia; 6 Medical Research and Education Center, Lomonosov Moscow State University, 27/10, Lomonosovsky Ave., 119192 Moscow, Russia; natalyadanilova@gmail.com (N.D.); malkovp@gmail.com (P.M.); dstambolsky@gmail.com (D.S.); 7 Research Institute of Human Morphology, 3, Tsyurupy Str., 117418 Moscow, Russia; bezuglovat@mail.ru (T.B.); balajancz@yandex.ru (V.B.); mnichmaxim@yandex.ru (M.M.)

**Keywords:** intracerebral hemorrhage, stroke, brain-derived neurotrophic factor, urokinase plasminogen activator, glutamate-induced neurotoxicity, microglia activation

## Abstract

Brain-derived neurotrophic factor (BDNF) is a classic neuroprotective and pro-regenerative factor in peripheral and central nervous tissue. Its ability to stimulate the restoration of damaged nerve and brain tissue after ischemic stroke and intraventricular hemorrhage has been demonstrated. However, the current concept of regeneration allows us to assert that one factor, even if essential, cannot be the sole contributor to this complex biological process. We have previously shown that urokinase-type plasminogen activator (uPA) complements BDNF activity and stimulates restoration of nervous tissue. Using a model of intracerebral hemorrhage in rats, we investigated the neurotrophic and neuroprotective effect of BDNF combined with uPA. The local simultaneous administration of BDNF and uPA provided effective neuroprotection of brain tissue after intracerebral hemorrhage, promoted survival of experimental animals and their neurological recovery, and decreased lesion volume. The study of cellular mechanisms of the observed neurotrophic effect of BDNF and uPA combination revealed both known mechanisms (neuronal survival and neurite growth) and new ones (microglial activation) that had not been shown for BDNF and uPA. Our findings support the concept of using combinations of biological factors with diverse but complementary mechanisms of action as a promising regenerative approach.

## 1. Introduction

Brain-derived neurotrophic factor (BDNF) is a classic neuroprotective and pro-regenerative factor in peripheral and central nervous tissue. Its ability to stimulate the restoration of damaged nervous tissue after ischemic stroke and intraventricular hemorrhage has been demonstrated [1,2]. Neutralizing BDNF in the secretome of mesenchymal stromal cells (MSC) with blocking antibodies or short hairpin RNA (shRNA) reduces the therapeutic effect of the secretome [3]. This confirms the indispensability of BDNF in the functioning and regeneration of nervous tissue. According to modern concepts, however, many proteins with different modalities (growth factors, cytokines, proteases, and matrix proteins) that compete with or complement each other are involved in the processes of development, functioning, and regeneration of tissues. Based on this fact, we showed that a combination of therapeutic genes with different modalities (BDNF and urokinase-type plasminogen activator (uPA)) is beneficial for peripheral nerve regeneration [4]. In our opinion, uPA is one of the most successful partners for BDNF for better stimulation of nervous tissue regeneration, because uPA is involved in a wide range of processes of nervous tissue ontogenesis and regeneration: navigation and outgrowth of neurites, fibrinolysis at the site of injury, and activation of growth factors, including BDNF [5,6,7]. The importance of the plasminogen activator system (uPA/uPAR, etc.) in the processes of nervous tissue regeneration is confirmed by upregulation of their expression in neural cells after injury [8,9].

Such outstanding pro-regenerative properties of BDNF and uPA could be applied for treating currently untreatable severe neurological conditions, such as intracerebral hemorrhage. A previously described bicistronic genetic construct, encoding human BDNF and uPA, efficiently stimulated restoration of the injured peroneal nerve [4]. Therefore, in the present study we explored the potential of a combination of human BDNF and uPA to stimulate brain tissue recovery after intracerebral hemorrhage.

## 2. Materials and Methods

### 2.1. Cell Culture

SH-SY5Y cells were obtained from ATCC (CRL-2266) and cultured in Dulbecco’s Modified Eagle Medium (DMEM)/F12 supplemented with 10% fetal bovine serum (FBS) and 1% penicillin-streptomycin (all from Gibco, Grand Island, NY, USA). HEK293T were obtained from ATCC (CRL-3216) and cultured in DMEM High Glucose medium supplemented with 1% penicillin-streptomycin with or without 10% FBS (all from Gibco, USA). Medium was changed every 3–4 days.

### 2.2. Preparation of Conditioned Medium Samples

Conditioned medium samples were obtained from HEK293T cells transfected with pVax1, pVax1-hBDNF [10], pVax1-huPA, or pNCure-fIntr [4], denoted as Control, BDNF, uPA, and BU (BDNF + uPA), respectively. For this endeavor, transfected HEK293T cells were cultured in serum-free DMEM High Glucose for 48 h. Then medium samples were removed, centrifuged for 10 min at 3000× *g* to remove cell debris, and concentrated ~50 times using a Centriprep Centrifugal Filter Unit (Merck, #4302, Tullagreen, Ireland) until a hBDNF concentration of 3.5 ng/µL (108 nM) was reached, confirmed using the Human Free BDNF Quantikine ELISA Kit (R&D Systems, #DBD00, Minneapolis, MN, USA). The huPA concentration in uPA and BU medium samples was adjusted to 0.4 ng/µL (8 nM), confirmed with the uPA (URK) Human ELISA Kit (Abcam, #ab119611, Waltham, MA, USA).

### 2.3. In Vitro Study of Neurotrophic Activity of BDNF, uPA, and Their Combination

To assess the direct neuroprotective activity of BDNF, uPA, and BU samples, we used a previously published in vitro model of glutamate-induced neurotoxicity [11]. This cellular model reflects one of the main causes of neuronal death during stroke: glutamate-induced neurotoxicity. For this endeavor, SH-SY5Y neuroblastoma cells were seeded in 48-well plates in complete growth medium at 40,000 cells/well in quadruplicates. On the next day, the medium was removed and samples of serum-free medium, control (C+) or containing 108 nM of hBDNF and/or 8 nM of huPA, with 100 mM L-glutamate [11] and 5 μM IncuCyte^®^ Caspase-3/7 Apoptosis Reagent (Essen Bioscience, #4440, Ann Arbor, MI, USA) were added. In the control group C- no L-glutamate, hBDNF or huPA were added to monitor the spontaneous cell death in serum-free medium. Glutamate causes a rapid increase in the cytosolic concentration of Ca2+ in SH-SY5Y cells (similarly to what happens in neurons), with subsequent caspase activation. The IncuCyte^®^ Caspase-3/7 Apoptosis Reagent, cleaved with activated caspase-3/7, stains nuclear DNA (green fluorescence). To monitor the death of SH-SY5Y cells, the plate was placed in the Incucyte^®^ ZOOM Live Cell Analysis System (Essen Bioscience), located inside a carbon dioxide (CO_2_) incubator. The time-lapse imaging of nine fields of vision (phase and green channel) of each well was performed at 1, 3, 6, 12, 24, 48, and 72 h after medium replacement.

To assess the ability of BDNF, uPA, and BU samples to stimulate neurite outgrowth, SH-SY5Y cells were seeded in 48-well plates in complete growth medium at 40,000 cells/well in quadruplicates. On the next day, the medium was removed and samples of Control medium or medium, containing 108 nM of hBDNF and/or 8 nM of huPA, were added. To monitor the neurite outgrowth of SH-SY5Y cells, the plate was placed in the Incucyte^®^ ZOOM Live Cell Analysis System (Essen Bioscience), located inside a CO_2_ incubator. The time-lapse imaging of nine fields of vision of each well was performed at 0, 6, and 24 h after medium replacement.

The micrographs were analyzed with ImageJ (version 1.41) software (National Institutes of Health, Bethesda, MD, USA) to calculate the percentage of living cells (compared with the initial cell number) and neurite outgrowth at each time point. Cell processes longer than two cell diameters were considered neurites.

### 2.4. Animals

The mature male Wistar rats used in this study were of between 3.0 and 3.5 months old and had standard weight characteristics. Animals were housed and used for experimental procedures in full compliance with Directive 2010/63/EU and the recommendations of the local Bioethical Committee of Lomonosov Moscow State University (permissions #3-2017, 29 October 2017 and #3.2, 16 January 2019).

### 2.5. Intracerebral Hemorrhage Modeling and Treatment

The study was carried out on a model of intracerebral hemorrhage into the inner capsule of the right hemisphere according to Makarenko [12]. The inner capsule contains many neural tracts that connect the cortex to the basal ganglia, nuclei of the spinal cord, and cranial nerves. Therefore, injury to the inner capsule causes various neurological deficits, and their severity correlates with brain injury severity.

The study included 70 animals that were randomly divided into five experimental groups: sham operated (SO), Control, BDNF, uPA, and BU (14 rats for each group). None of the rats were excluded from the study. To model the hemorrhage, rats were anesthetized with a solution containing 2% Zoletil^®^ 50 (VirBac, Carros Cedes, France) and 1.5% xylazine (InterChemie, Venray, the Netherlands) in saline at a dose of 1 mL/kg body weight and positioned in a stereotaxic frame. The scalp and skull aponeurosis were dissected, and the cranium was perforated (bregma −2.0 mm, lateral 3.5 mm) [13]. Local brain tissue was destroyed and the superior cerebral veins on the corresponding side were injured to model a bleed. To standardize the volume of spilled blood, 20 µL of autologous blood obtained from the hypoglossal vein were slowly injected into the injury focus. In 5 min, the blinded investigator injected 20 µL of an appropriate concentrated medium sample and the wound was sutured. The animals from the SO group underwent cranium perforation without any subsequent brain damage or blood/medium sample injections.

### 2.6. Neurological Status Assessment

The animals were observed for 14 days following the intracerebral hemorrhage, with daily recording of rat deaths and neurological status assessment at 3 and 10 days following the hemorrhage (Stroke-index McGraw scale, modified for rodents by Ganushkina) [14,15]. Briefly, “visually healthy animals” had no signs of neurological deficit. The animals with signs of lethargy, limb weakness, tremor, ptosis, and/or semi-ptosis were considered “slightly affected”. The animals demonstrating signs of paresis and/or paralysis of the limbs, impaired coordination, or in a coma were considered “severely affected”. The researchers conducting the neurological testing were blinded to each rat’s treatment.

### 2.7. Magnetic Resonance Imaging (MRI)

MRIs were obtained 11 days following the intracerebral hemorrhage using the Clinscan 7T system (Bruker Biospin, Billerica, MA, USA) equipped with the rat brain surface coil with TurboS Spin Echo sequence and fat suppression. The coronary projections were acquired using the following parameters: TR (Repetition time) = 5220 ms; TE (Time to Echo) = 53 ms; echo train length = 9; base resolution 230 × 320; FoV (Field-of-view) = 32 × 40 mm; slice thickness = 0.5 mm; spacing between the slices = 0.75 mm. The transversal projections were acquired using the following parameters: TR = 4000 ms; TE = 40 ms; echo train length = 9; base resolution 288 × 320; FoV = 40 × 40 mm; slice thickness = 0.5 mm; spacing between the slices = 0.6 mm.

### 2.8. Histochemistry and Immunohistochemistry

For histological studies, the rats were euthanized on day 14 following the intracerebral hemorrhage by exposure to gradually increasing concentrations of CO_2_. The brain was removed, fixed in 4% formaldehyde solution, and embedded in paraffin. The brain slices containing the injury focus were dewaxed and antigens were unmasked. Some of the slices were stained with hematoxylin-eosin, cresyl violet (Nissl stain), or Perls Prussian blue (Perls stain). A combination of these stains allows assessing the various aspects of morphological changes within an injured brain: the size of the lesion, neuronal status, leukocyte infiltration rate, and the size of hemosiderin deposits.

For immunohistochemical staining, the slices were treated with 50 mM ammonium acetate solution and blocked with 10% goat serum to reduce background fluorescence. After blocking, they were incubated with antibodies to CD68 (Abcam, #ab125212, USA) and CD163 (Abcam, #ab182422, USA), followed by incubation with fluorescently labeled secondary goat-anti-rabbit antibodies (Invitrogen, #A11034, Eugene, OR, USA). The nuclei were stained with 4′, 6-diamidino-2-phenylindole (DAPI) solution (Sigma, #MBD0015-10ML, St. Louis, MO, USA). The specimens were examined with a Leica DM600 microscope equipped with DFC360 FX and DFC420C camera (Leica Microsystems GmbH, Wetzlar, Germany), using representative fields of view to obtain photographs. Image processing and analysis were performed using LasX software (Leica Microsystems GmbH, Germany) and FiJi.

### 2.9. Statistical Analysis

Statistical analysis was performed using SigmaPlot11.0 software (Systat Software, Inc., Erkrath, Germany). Numerical data was assessed for normality of distribution using the Kolmogorov–Smirnov test. Differences between the treatment and control groups were analyzed using Student’s *t*-test or analysis of variance (ANOVA) on ranks (Dunn’s test), depending on whether the data were normally distributed. Data are expressed as the mean ± standard deviation or the median (25%; 75%) depending on the test used. We considered differences to be significant when *p* < 0.05.

To analyze the categorical data (neurological outcome after stroke), Fisher’s exact test was used: BDNF, uPA and BU groups were compared to the Control group pairwise. Since Fisher’s exact test only supports 2 × 2 massives (χ2 is not applicable for small groups), the proportions of animals were combined into two groups: “visually healthy animals” + “slightly affected”, “severely affected” + “dead”. Different points in time were compared separately

## 3. Results

### 3.1. uPA Increases the Neuroprotective Activity of BDNF in a Model of Intracerebral Hemorrhage

**Figure 1:**
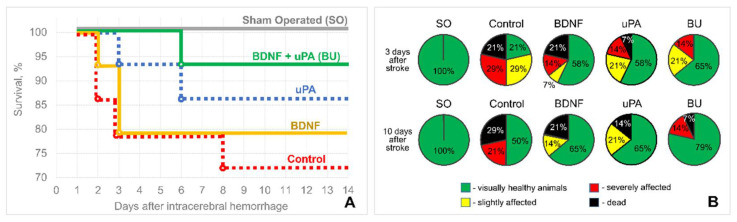
Animal survival (**A**) and the neurological percentage structure (**B**) of experimental groups at 3 and 10 days following the intracerebral hemorrhage (according to the neurological testing data). BDNF, brain-derived neurotrophic factor; BU, BDNF + uPA; SO, sham-operated; uPA, urokinase-type plasminogen activator.

We studied the protective and pro-regenerative properties of BDNF and its combination with uPA in a rat model of intracerebral hemorrhage. We found that conditioned medium containing 108 nM of hBDNF (3 ng/µL) slightly increased the neurological outcome of the experimental rats but did not influence their survival. Supplementing the BDNF-containing conditioned medium with 8 nM of uPA (0.4 ng/µL) dramatically increased the survival of the experimental animals and decreased the severity of the observed neurological deficits (Figure 1). Thus, 64% and 79% of animals had no visible neurological deficits (looked healthy) at 3 and 10 days following the intracerebral hemorrhage in the BU group. In the Control group, only 21% and 50% of animals looked healthy at 3 and 10 days following the IHM. However, there were no statistical differences between the BU and Control groups: *p* = 0.052 at 3 days and *p* = 0.147 at 10 days after stroke (n = 14; two-sided Fisher’s exact test). uPA had effect similar to BDNF: it stimulated the survival of rats (86% at 10 days after intracerebral hemorrhage). No deaths were observed within the first 3 days and total lethality during the entire experiment (14 days) in the BU group was 7% (1 of 14), while in the Control group the lethality reached 29% (4 of 14).

**Figure 2:**
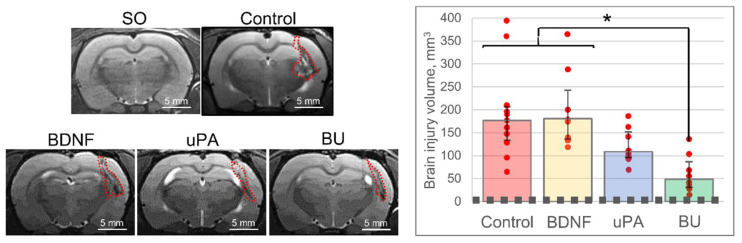
The results of magnetic resonance imaging of brains 11 days following the intracerebral hemorrhage. On the images, the dashed red line corresponds to the volume of the brain lesion focus in sham-operated (SO) animals. Red dots on the diagram correspond to the result of individual measurements. The data are presented as the median (25%; 75%). *—H (3, 28) = 16.424, *p* = 0.001. BDNF, brain-derived neurotrophic factor; BU, BDNF + uPA; uPA, urokinase-type plasminogen activator.

The data of animal survival and neurological assessment correlated with the MRI findings. In the BU group, the brain injury volume was significantly less than in the Control group [H (3, 28) = 16.424, *p* = 0.001]: 48.5 (31.0; 86.7) mm^3^ versus 176.6 (133.8; 206.3) mm^3^ (Figure 2). According to the MRI data, in the BDNF and uPA groups, the brain injury volume reached 180.7 (136.1; 242.8) and 108.6 (96.1; 151.9) mm^3^, respectively, with no significant difference compared with the Control group.

**Figure 3:**
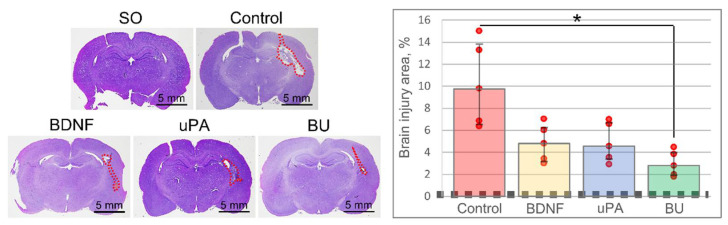
The histological examination of brain slices. Hematoxylin-eosin staining reveals the site of damage (red dashed curved line). On the diagram: the dashed line corresponds to the area of the brain lesion in sham-operated (SO) animals and the red dots—to the result of individual measurements. The data are presented as the median (25%; 75%). *—H (3, 12) = 11.046, *p* = 0.011. BDNF, brain-derived neurotrophic factor; BU, BDNF + uPA; uPA, urokinase-type plasminogen activator.

Hematoxylin-eosin stain of the brain slices revealed that the injury volume was significantly less in the BU group than in the Control group [H (3, 12) = 11.046, *p* = 0.011]: 2.8% (2.0%; 3.9%) versus 9.8% (6.6%; 14.3%). In the BDNF and uPA groups, the volume of the injury locus was 4.8% (3.1%; 6.7%) and 4.6% (3.5%; 6.8%), respectively, with no significant difference compared with the Control group (Figure 3). These data correlate with the MRI and neurological findings.

Hematoxylin-eosin and Nissl staining of the brain slices confirmed the presence of microscopic signs of brain tissue damage: necrosis, ischemia, edema, glial scar, neutrophilic infiltration, and blood stasis in the capillary bed (Figures S1 and S2). The signs of damage were more prominent in the Control group, but quantitative analysis was not performed. Perls Prussian blue staining demonstrated deposits of hemosiderin (blue) within the brain tissue adjacent to the injury focus in all groups except the Control (Figure S2). We suppose this pattern indicates that in the Control group, the primary lesion had expanded and thus engulfed the deposits of hemosiderin.

### 3.2. A Combination of BDNF and uPA Potentiates Phagocytic Activity of Monocytes/Macrophages in the Penumbra

**Figure 4:**
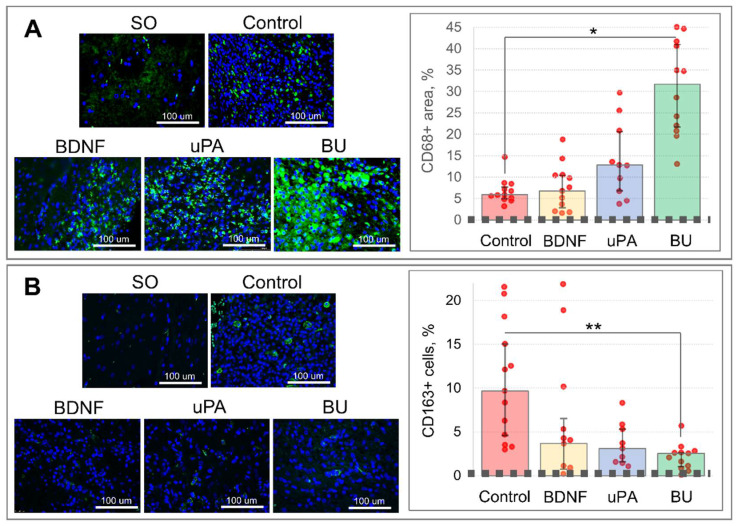
The results of immunohistochemical staining of brain slices 14 days following the intracerebral hemorrhage: (**A**)—CD68 and (**B**)—CD163. On the graphs, the dashed lines correspond to the CD68- or CD163-stained area in the brain slices of sham-operated (SO) animals and the red dots—to the result of individual measurements. The data are presented as the median (25%; 75%). *—H (3, 36) = 25.439, *p* = 0.001. **—H (3, 32) = 14.998, *p* = 0.002. BDNF, brain-derived neurotrophic factor; BU, BDNF + uPA; uPA, urokinase-type plasminogen activator.

Immunohistochemical staining of the brain slices for CD68, the marker of proinflammatory M1 microglia and lysosomal activity, showed its significantly higher expression in the brain sections of the BU group [H (3, 36) = 25.439, *p* = 0.001]: 31.7% (21.4%; 41.3%) of the area stained for CD68 versus 5.9% (5.0%; 7.7%) in the Control group (Figure 4A). In the BDNF and uPA groups, the CD68-stained area reached 6.8% (2.8%; 10.4%) and 12.8% (6.8%; 20.6%), respectively, with no significant difference compared with the Control group. Although there was not a significant difference in CD68 staining between the BDNF and uPA groups, uPA is apparently able to stimulate the activation of residual microglia or attract peripheral blood monocytes/macrophages.

Staining for CD163, the marker of M2 microglia and hemoglobin-activated macrophages, showed a significant decrease in the brain infiltration by CD163+ cells in the BU group [H (3, 32) = 14.998, *p* = 0.002]: 2.5% (1.0%; 2.6%) versus 9.7% (4.3%; 15.8%) in the Control group (Figure 4B). A similar decrease in CD163+ cells was observed in the BDNF and uPA groups: 3.7% (0.5%; 7.8%) and 3.1% (1.5%; 5.5%), respectively; these values did not differ significantly compared with the Control group.

### 3.3. A Combination of BDNF and uPA Stimulates Survival and Neurite Outgrowth of SH-SY5Y Cells

**Figure 5:**
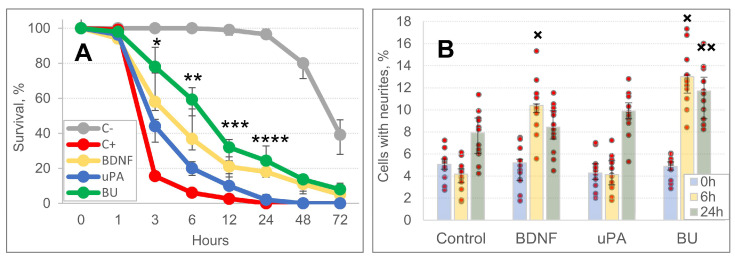
Brain-derived neurotrophic factor (BDNF), urokinase-type plasminogen activator (uPA) and their combination support the survival of SH-SY5Y neuroblastoma cells under glutamate-excitotoxic conditions (**A**) and stimulates neuritogenesis in SH-SY5Y cells (**B**). In (**A**), BDNF, uPA, and their combination (BU) significantly differ from the positive control (C+) group within the time range of 3–24 h: [*—H (3, 44) = 39.548, *p* = 0.001; **—H (3, 44) = 32.954, *p* = 0.001; ***—H (3, 44) = 24.681, *p* = 0.001; ****—H (3, 44) = 40.080, *p* = 0.001]. In (B), ×—H (3, 44) = 35800, *p* = 0.001; ××—H (3, 44) = 12.879, *p* = 0.005. Red dots on the diagram correspond to the result of individual measurements. All the comparisons were made versus the Control group value at the corresponding time point.

To study the mechanism of neuroprotective activity of BDNF and uPA, we evaluated their ability to prevent cell death in neurotoxic conditions and stimulate neurite outgrowth. Samples of medium, containing hBDNF (108 nM), huPA (8 nM) or their combination, supported the survival of SH-SY5Y cells (up to 24–48 h) under the conditions of glutamate-induced neurotoxicity much more efficiently compared with the positive control (C+) group [*—H (3, 44) = 39.548, *p* = 0.001; **—H (3, 44) = 32.954, *p* = 0.001; ***—H (3, 44) = 24.681, *p* = 0.001; ****—H (3, 44) = 40.080, *p* = 0.001] (Figure 5A, Figures S3 and S4). In addition to glutamate-mediated toxicity, spontaneous death of SH-SY5Y cells was also observed. It increased significantly 24 h after the in vitro experiment began, specifically in the negative control group (C-, serum-free medium without L-glutamate).

The study of the dynamics of SH-SY5Y neurite outgrowth within 24 h showed spontaneous neurite growth in all experimental groups. BDNF and BU stimulated neurite outgrowth significantly better than the Control medium sample 6 h after the experiment began [H (3, 44) = 35800, *p* = 0.001] (Figure 5B and Figure S5). After 6 h in the BU group, 13.0% (11.5%; 13.1%) of cells had neurites, whereas only 4.2% (3.4%; 4.7%) of cells in the Control group had neurites. After 24 h, only the BU group differed from the Control [H (3, 44) = 12.879, *p* = 0.005], the other groups did not: 7.6% (6.2%; 9.2%) for Control, 8.5% (7.2%; 9.9%) for BDNF, 9.7% (9.1%; 10.6%) for uPA, and 11.2% (9.1%; 13.0%) for BU.

## 4. Discussion

In the current study, we aimed to assess the potential therapeutic benefit of a classic neurotrophin (BDNF) alone and in combination with uPA to stimulate neuroprotection and brain recovery after stroke. BDNF or uPA alone showed a moderate therapeutic effect on the survival of experimental animals, their neural recovery, and lesion volume shrinkage. However, complementing BDNF with uPA significantly increased the survival of experimental animals after intracerebral hemorrhage and reduced the severity of the observed neurological deficits. The mechanisms of this phenomenon are not well understood, but we hypothesize that this may be due to the pleiotropic activity of uPA [5,6,7] and its ability to complement the neurotrophic activity of BDNF.

BDNF is a classic neurotrophic factor that supports functioning of nervous tissue as well as stimulates neuronal survival and neurite outgrowth [1,2,10]. However, tissue- and urokinase-type plasminogen activators (tPA and uPA) are also essential for nervous tissue regeneration, as confirmed by previously published data that the expression of plasminogen activators is upregulated after nervous tissue injury and that animals with knocked-out genes of the plasminogen-activator system demonstrate delayed and incomplete recovery of nervous tissue [16,17]. Thus, the uPA/plasminogen system is known to activate growth factors, including BDNF, trigger fibrinolysis, stimulate angiogenesis, and participate in the navigation of regenerating nerve fibers [5,6,7].

These uPA abilities may underlie the observed stimulation of brain tissue regeneration. Some of the mechanisms have been confirmed in our work. Specifically, we showed that medium containing human BDNF, uPA, and especially their combination (BU) stimulated the survival of SH-SY5Y cells in a model of glutamate-induced neurotoxicity, while BDNF or uPA per se were less effective. The combination of BDNF and uPA also provided more notable neuritogenesis in SH-SY5Y cell culture compared with BDNF or uPA alone. This difference is presumably due to the ability of the uPA/plasminogen system to convert proBDNF to the mature form, which has a predominant affinity for the tropomyosin receptor kinase B (trkB) (but not for the proapoptotic p75 receptors) that stimulates the survival of neural cells and enhances their neuritogenesis [18].

Other mechanisms may also be involved in nervous tissue regeneration supported by BDNF and uPA administration. We found a massive upregulation of CD68 staining in the ischemic penumbra of rats treated with uPA or a combination of uPA and BDNF. CD68 is a marker of phagocytosis and autophagy and a marker of primed (preactivated) microglia or activated peripheral blood monocytes/macrophages [19,20]. Interestingly, we found that CD68 expression correlated with better survival and neurological recovery of experimental animals. This outcome was presumably due to the activation of the phagocytic function in microglia/monocytes/macrophages that promoted early resorption of blood and necrotic masses and prevented the expansion of the lesion focus. Previously, this mechanism of brain tissue recovery (restricted microglial activation) had been assumed [20] but had not been confirmed experimentally. In contrast, hyperactivation of microglia increases inflammation, expands injury area and aggravates the outcome [21].

Based on our data, we cannot conclude whether this effect is due to uPA activity per se or the combined effect of BDNF and uPA. The following hypothesis is supported by published data: BDNF and/or the uPA/plasminogen system stimulates the invasion of monocytes/macrophages [22], promotes the maturation of macrophages [23,24,25], and contributes to the progression of chronic inflammation in the brain [26]. Prolonged expression of these proteins may contribute to brain injury and dysfunction; however, in the model of intracerebral hemorrhage, a pulse injection of BDNF and uPA and subsequent activation of microglia/monocytes/macrophages was beneficial.

uPA is a classic plasminogen activator and is used to recanalize clotted blood vessels, so it can be said that using uPA to treat intracerebral hemorrhage can re-initiate bleeding. However, there is no contradiction: uPA is a pleiotropic molecule (protease, ligand, and transcription factor), and its predominant mechanism of action strongly depends on its concentration [27,28]. Here, we used 8 nM uPA, which is 30-times lower than the concentration for therapeutic fibrinolysis (>250 nM) [29]. Moreover, this is a proof-of-concept study, and further studies can use forms of uPA with altered activity or its functional analogs.

As a plasmid encoding hBDNF and huPA was effective for treating injured nerves [4], we considered it would stimulate brain recovery in the intracerebral hemorrhage model. However, we later abandoned this option because the production of a recombinant protein during gene therapy takes about 24–48 h, but a stroke, unlike nerve damage, is an emergent condition and requires an urgent intervention. Moreover, we encountered extremely low efficiency of gene delivery (plasmids, lentiviral particles, and extracellular vesicles) into the ischemic penumbra after inducing the intracerebral hemorrhage (data not shown).

In our study, we used young and healthy experimental animals, because we aimed to answer the pivotal questions: is it possible to treat intracerebral hemorrhages, and what pathogenetic mechanisms could be impacted to provide neuroprotection and stop the progression of secondary damage. We have shown that it is fundamentally possible to protect and to regenerate brain tissue after intracerebral hemorrhages of various natures, in particular, those caused by COVID-19, and that the application of BDNF and uPA combination looks promising for this purpose.

The results obtained may serve as the first step towards the development of an effective therapeutic approach for treating the intracerebral hemorrhages. However, further studies regarding the route and regimen of administration as well as the therapeutic “time window” and effective dose requires studies in aged animals or in animals with cardiovascular diseases, since age, as well as concomitant diseases and habitual intoxications, are not only the causes of the cerebrovascular disorders, but also largely determine the effectiveness of recovery processes after damage [30,31].

The other important observation of this study is that a combination of bioactive compounds with different modalities is more effective than single molecules. This finding confirms the concept of the complexity of regeneration processes and the idea of stimulating them using mixtures of bioactive compounds.

## Data Availability

Not applicable.

## Supplementary Materials

The following supporting information can be downloaded at: https://www.mdpi.com/article/10.3390/biomedicines10061346/s1, Figure S1: Histological signs of brain tissue damage (hematoxylin-eosin): In (A and B), a necrosis focus restrained by neutrophil infiltration (NI) and a glial scar (GS); Figure S2: Histological examination of brain slices. Perls stain reveals hemosiderin deposits (blue grains). Nissl stains reflects the functional state of neurons in the penumbra: red arrows for alive though hypoxic neurons, black arrows for dead neurons (neuron shadows); Figure S3: Samples of images (raw data) obtained during the study of neuroprotective activity of BDNF, uPA and BU medium samples in the model of in vitro glutamate-mediated excitotoxicity. Green stain marks dead cells (apoptosis); Figure S4: Brain-derived neurotrophic factor (BDNF), urokinase-type plasminogen activator (uPA) and their combination support the survival of SH-SY5Y neuroblastoma cells under glutamate-excitotoxic conditions (scatter plots); Figure S5: Samples of images (raw data) obtained during the study of ability of BDNF, uPA and BU medium samples to stimulate neuritogenesis.

## References

[1] Ahn S.Y., Sung D.K., Kim Y.E., Sung S., Chang Y.S., Park W.S. (2021). Brain-derived neurotropic factor mediates neuroprotection of mesenchymal stem cell-derived extracellular vesicles against severe intraventricular hemorrhage in newborn rats. Stem Cells Transl. Med..

[2] Liu W., Wang X., O’Connor M., Wang G., Han F. (2020). Brain-Derived Neurotrophic Factor and Its Potential Therapeutic Role in Stroke Comorbidities. Neural Plast..

[3] Lopatina T., Kalinina N., Karagyaur M., Stambolsky D., Rubina K., Revischin A., Pavlova G., Parfyonova Y., Tkachuk V. (2011). Adipose-derived stem cells stimulate regeneration of peripheral nerves: BDNF secreted by these cells promotes nerve healing and axon growth de novo. PLoS ONE.

[4] Karagyaur M., Rostovtseva A., Semina E., Klimovich P., Balabanyan V., Makarevich P., Popov V., Stambolsky D., Tkachuk V. (2020). A Bicistronic Plasmid Encoding Brain-Derived Neurotrophic Factor and Urokinase Plasminogen Activator Stimulates Peripheral Nerve Regeneration After Injury. J. Pharmacol. Exp. Ther..

[5] Semina E., Rubina K., Sysoeva V., Rysenkova K., Klimovich P., Plekhanova O., Tkachuk V. (2016). Urokinase and urokinase receptor participate in regulation of neuronal migration, axon growth and branching. Eur. J. Cell Biol..

[6] Sisson T.H., Nguyen M.H., Yu B., Novak M.L., Simon R.H., Koh T.J. (2009). Urokinase-type plasminogen activator increases hepatocyte growth factor activity required for skeletal muscle regeneration. Blood.

[7] Rivellini C., Dina G., Porrello E., Cerri F., Scarlato M., Domi T., Ungaro D., Del Carro U., Bolino A., Quattrini A. (2012). Urokinase plasminogen receptor and the fibrinolytic complex play a role in nerve repair after nerve crush in mice, and in human neuropathies. PLoS ONE.

[8] Hayden S.M., Seeds N.W. (1996). Modulated expression of plasminogen activator system components in cultured cells from dissociated mouse dorsal root ganglia. J. Neurosci..

[9] Yepes M., Woo Y., Martin-Jimenez C. (2021). Plasminogen Activators in Neurovascular and Neurodegenerative Disorders. Int. J. Mol. Sci..

[10] Karagyaur M., Dyikanov D., Makarevich P., Semina E., Stambolsky D., Plekhanova O., Kalinina N., Tkachuk V. (2015). Non-viral transfer of BDNF and uPA stimulates peripheral nerve regeneration. Biomed. Pharmacother..

[11] Nampoothiri M., Reddy N.D., John J., Kumar N., Kutty Nampurath G., Rao Chamallamudi M. (2014). Insulin blocks glutamate-induced neurotoxicity in differentiated SH-SY5Y neuronal cells. Behav. Neurol..

[12] Makarenko A.N., Kositsyn N.S., Pasikova N.V., Svinov M.M. (2002). Simulation of local cerebral hemorrhage in different brain structures of experimental animals [russian]. Zhurnal Vyss. Nervn. Deiatelnosti Im. IP Pavlov..

[13] Paxinos G., Watson C. (2007). The Rat Brain in Stereotaxic Coordinates.

[14] McGraw C.P., Pashayan A.G., Wendel O.T. (1976). Cerebral infarction in the Mongolian gerbil exacerbated by phenoxybenzamine treatment. Stroke.

[15] Gannushkina I.V. (2000). Mozgovoe krovoobrashchenie pri razlichnykh vidakh tsirkuliatornoĭ gipoksii mozga [Cerebral circulation in different types of brain hypoxia]. Vestn. Ross. Akad. Meditsinskikh Nauk.

[16] Diaz A., Merino P., Manrique L.G., Ospina J.P., Cheng L., Wu F., Jeanneret V., Yepes M. (2017). A Cross Talk between Neuronal Urokinase-type Plasminogen Activator (uPA) and Astrocytic uPA Receptor (uPAR) Promotes Astrocytic Activation and Synaptic Recovery in the Ischemic Brain. J. Neurosci..

[17] Klimovich P.S., Semina E.V., Karagyaur M.N., Rysenkova K.D., Sysoeva V.Y., Mironov N.A., Sagaradze G.D., Az’muko A.A., Popov V.S., Rubina K.A. (2020). Urokinase receptor regulates nerve regeneration through its interaction with α5β1-integrin. Biomed. Pharmacother..

[18] Gray K., Ellis V. (2008). Activation of pro-BDNF by the pericellular serine protease plasmin. FEBS Lett..

[19] Li J.W., Zong Y., Cao X.P., Tan L., Tan L. (2018). Microglial priming in Alzheimer’s disease. Ann. Transl. Med..

[20] Witcher K.G., Eiferman D.S., Godbout J.P. (2015). Priming the inflammatory pump of the CNS after traumatic brain injury. Trends Neurosci..

[21] Karagyaur M., Dzhauari S., Basalova N., Aleksandrushkina N., Sagaradze G., Danilova N., Malkov P., Popov V., Skryabina M., Efimenko A. (2021). MSC Secretome as a Promising Tool for Neuroprotection and Neuroregeneration in a Model of Intracerebral Hemorrhage. Pharmaceutics.

[22] Fleetwood A.J., Achuthan A., Schultz H., Nansen A., Almholt K., Usher P., Hamilton J.A. (2014). Urokinase plasminogen activator is a central regulator of macrophage three-dimensional invasion, matrix degradation, and adhesion. J. Immunol..

[23] Menshikov M.Y., Stafeev I.S., Zubkova E.S., Beloglazova I.B., Ratner E.I., Dergilev K.V., Parfyonova E.V. (2019). The Role of Urokinase, Tumor Necrosis Factor, and Matrix Metalloproteinase-9 in Monocyte Activation. Bull. Exp. Biol. Med..

[24] Mizoguchi Y., Kato T.A., Seki Y., Ohgidani M., Sagata N., Horikawa H., Yamauchi Y., Sato-Kasai M., Hayakawa K., Inoue R. (2014). Brain-derived neurotrophic factor (BDNF) induces sustained intracellular Ca^2+^ elevation through the up-regulation of surface transient receptor potential 3 (TRPC3) channels in rodent microglia. J. Biol. Chem..

[25] Paland N., Aharoni S., Fuhrman B. (2013). Urokinase-type plasminogen activator (uPA) modulates monocyte-to-macrophage differentiation and prevents Ox-LDL-induced macrophage apoptosis. Atherosclerosis.

[26] Cunningham O., Campion S., Perry V.H., Ohgidani M., Sagata N., Horikawa H., Yamauchi Y., Sato-Kasai M., Hayakawa K., Inoue R. (2009). Microglia and the urokinase plasminogen activator receptor/uPA system in innate brain inflammation. Glia.

[27] Ge Y., Elghetany M.T. (2003). Urokinase plasminogen activator receptor (CD87): Something old, something new. Lab. Hematol..

[28] Rysenkova K.D., Klimovich P.S., Shmakova A.A., Karagyaur M.N., Ivanova K.A., Aleksandrushkina N.A., Tkachuk V.A., Rubina K.A., Semina E.V. (2020). Urokinase receptor deficiency results in EGFR-mediated failure to transmit signals for cell survival and neurite formation in mouse neuroblastoma cells. Cell. Signal..

[29] Gurewich V. (1987). Experiences with pro-urokinase and potentiation of its fibrinolytic effect by urokinase and by tissue plasminogen activator. J. Am. Coll. Cardiol..

[30] Popa-Wagner A., Petcu E.B., Capitanescu B., Hermann D.M., Radu E., Gresita A. (2020). Ageing as a risk factor for cerebral ischemia: Underlying mechanisms and therapy in animal models and in the clinic. Mech. Ageing Dev..

[31] Popa-Wagner A., Dumitrascu D.I., Capitanescu B., Petcu E.B., Surugiu R., Fang W.H., Dumbrava D.A. (2020). Dietary habits, lifestyle factors and neurodegenerative diseases. Neural Regen. Res..

