# Supplementary material for: Urokinase-Type Plasminogen Activator Enhances the Neuroprotective Activity of Brain-Derived Neurotrophic Factor in a Model of Intracerebral Hemorrhage
Source: Biomedicines. 2022 Jun 8;10(6):1346. doi: 10.3390/biomedicines10061346 (PMC9220139; doi:10.3390/biomedicines10061346)
Supplement: Supplementary file 1 [file biomedicines-10-01346-s001.zip › biomedicines-1738940-supplementary.pdf]

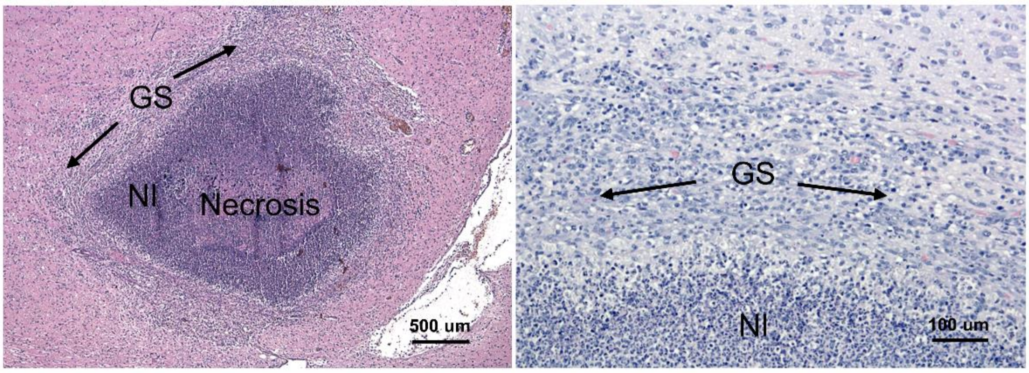

Figure S1. Histological signs of brain tissue damage (hematoxylin-eosin): In (A and B), a necrosis focus restrained by neutrophil infiltration (NI) and a glial scar (GS).

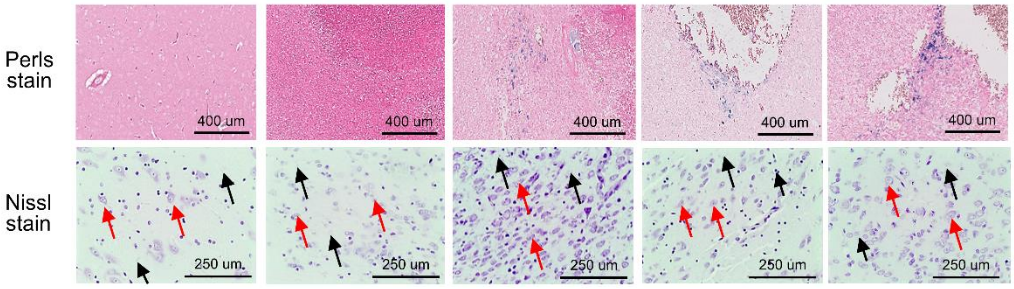

Figure S2. Histological examination of brain slices. Perls stain reveals hemosiderin deposits adjacent to the hemorrhage site (blue grains). Nissl stains reflects the functional state of neurons in the penumbra: red arrows for alive though hypoxic neurons, black arrows for dead neurons (neuron shadows).

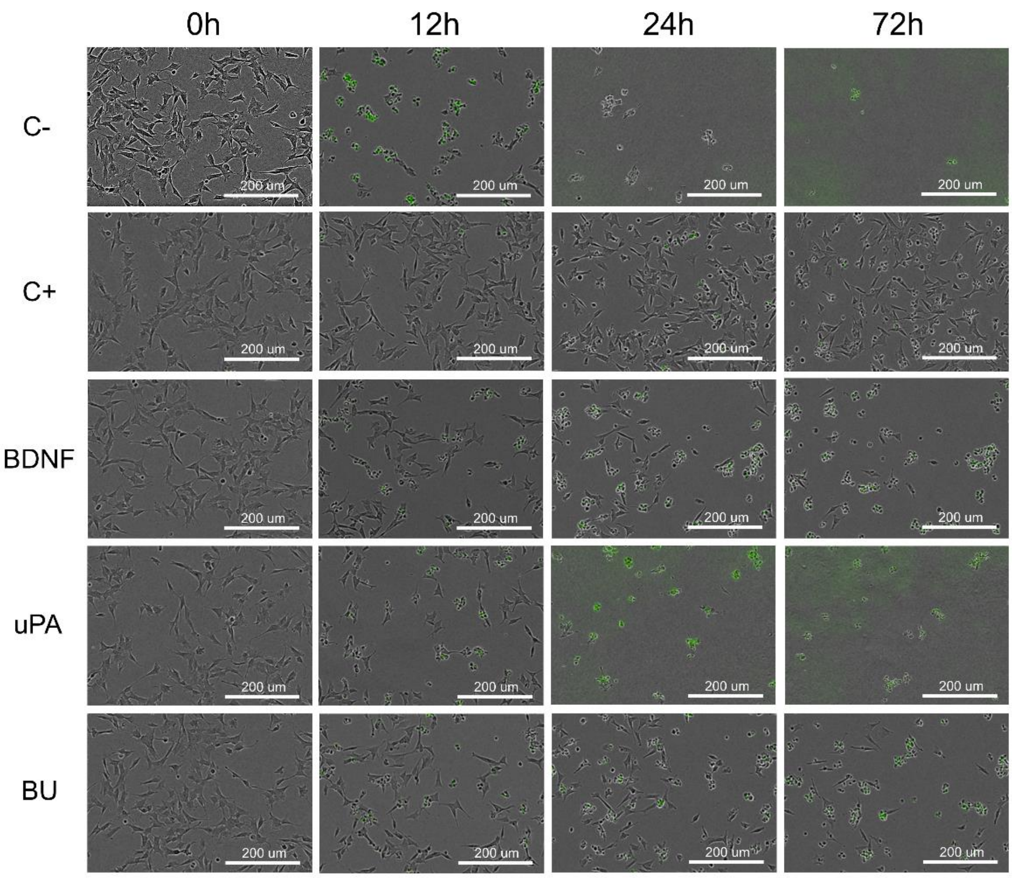

Figure S3. Samples of images (raw data) obtained during the study of neuroprotective activity of BDNF, uPA and BU medium samples in the model of in vitro glutamate-mediated excitotoxicity. Green stain marks dead cells (apoptosis).

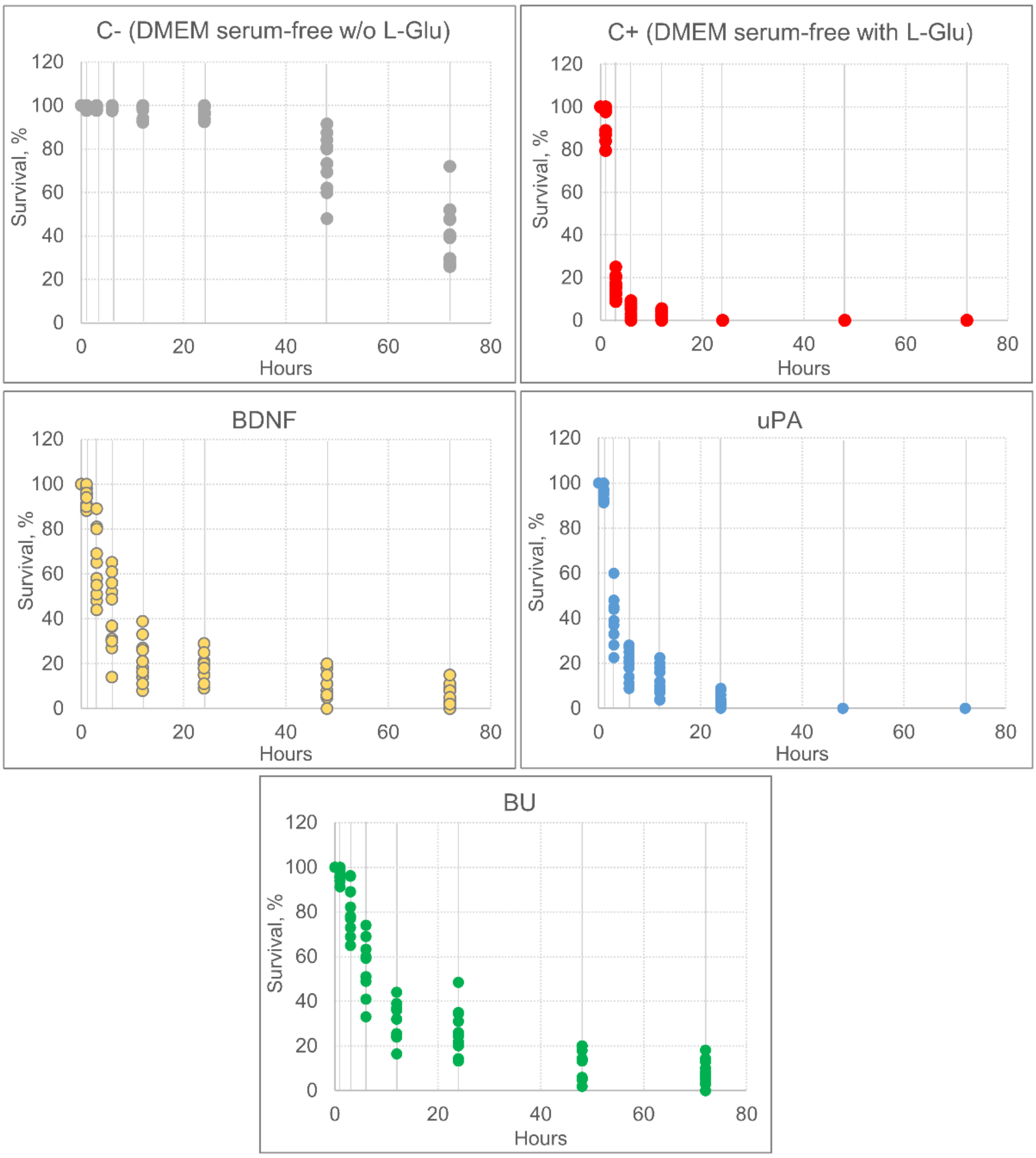

Figure S4. Brain-derived neurotrophic factor (BDNF), urokinase-type plasminogen activator (uPA) and their combination support the survival of SH-SY5Y neuroblastoma cells under glutamate-excitotoxic conditions (scatter plots).

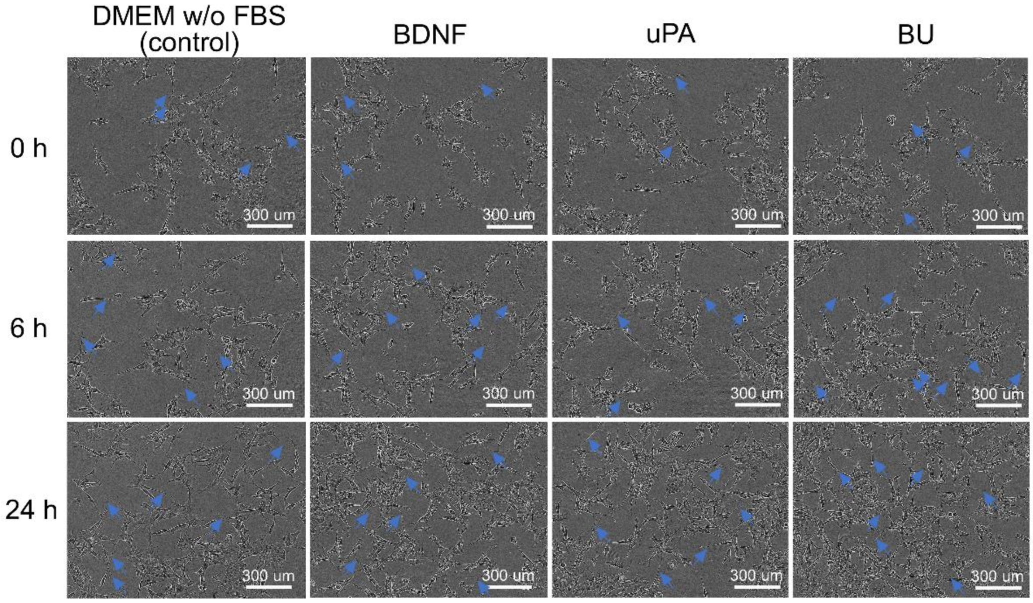

Figure S5. Samples of images (raw data) obtained during the study of ability of BDNF, uPA and BU medium samples to stimulate neuritogenesis. Blue arrows mark the neurites.
